# The efficacy of laser therapy in patients with facial palsy: A protocol for systematic review and meta-analysis

**DOI:** 10.1097/MD.0000000000021665

**Published:** 2020-08-21

**Authors:** Jung-Hyun Kim, Yeon-Cheol Park, Byung-Kwan Seo, Yong-Hyeon Baek, Bonhyuk Goo, Sang-Soo Nam

**Affiliations:** a Department of Acupuncture & Moxibustion, Kyung Hee University Hospital at Gangdong, Dongnam-ro, Gangdong-gu; b Department of Acupuncture & Moxibustion Medicine, College of Korean Medicine, Kyung Hee University, Kyungheedae-ro, Dongdaemun-gu, Seoul, Republic of Korea.

**Keywords:** facial palsy, facial paralysis, laser therapy, laser treatment, systematic review

## Abstract

**Background:**

Facial palsy involves paralysis of any structure affected by the facial nerve and affects facial appearance. Face palsy can result from congenital, idiopathic, neoplastic, infection-related, traumatic, malignant, diabetic, iatrogenic, and other inflammatory causes. Numerous studies have suggested that laser treatment is beneficial for managing facial palsy. The objectives of this review were to examine the effects of laser therapy in hastening recovery from long-term morbidity due to facial palsy.

**Methods:**

We will conduct a systematic analysis of controlled trials reviewing the efficacy of any laser therapy designed to treat facial palsy in patients. We will search multiple electronic databases, trial registries, and bibliographies and will contact authors to identify missing study details. We will use systematic review software to independently filter studies and extract available data and then will summarize characteristics of the study populations, interventions, comparators, outcomes, and quality/risk of bias. Primary outcomes will be categorized into paralysis score, physical facial disability index (PFDI), social facial disability index (SFDI), and recovery rate of supracordal/infracordal lesions. Secondary outcomes will be considered based on study findings. Planned trial subgroup analyses will determine whether the participants had a chronic condition, the type of comparator (i.e., placebo/sham vs. usual care), and study quality/risk of bias.

**Results:**

This review intends to establish evidence for laser therapies in improving recovery rates, particularly among patients with facial palsy.

**Conclusion:**

Its findings will be beneficial to clinicians and patients seeking innovative and effective ways to manage facial palsy and accompanying sequelae.

## 1 Introduction

The face is considered psychologically the most important part of the body and an important component of self-concept.^[1]^ Facial nerve paralysis is a common problem that affects appearance and involves paralysis of any structure innervated by the facial nerve. Lesions of the facial nerve can result in partial or full paralysis of 1 side of the face with impaired facial movement and diminished facial expression, which interfere with interactions with others and face-to-face communications.^[2]^ In addition, patients with unilateral or bilateral facial paralysis have difficulties eating, drinking, and speaking, which subsequently leads to impairment in activities of daily living as well as social and psychological difficulties, including anxiety, depression, social isolation, and lower self-esteem.^[3]^

Facial paralysis can result from congenital, idiopathic, neoplastic, iatrogenic, infectious, traumatic, viral (herpes zoster), malignant, diabetic, polyneuropathic, or inflammatory causes.^[4]^ Upon physical examination, the patient may be unable to raise the eyebrow or tightly close the eyelid on the affected side. Moreover, the nasolabial fold is typically absent, and the mouth may be drawn toward the unaffected side. Patients may drool from the affected side because of an inability to keep the mouth closed.^[5]^

Electrotherapy, massage, facial exercises, and biofeedback are various physical therapy modalities used for treatment of facial palsy, with a focus on exercise therapy. The aim of these modalities is to increase muscle and nerve function through exercise or electrotherapy. Furthermore, thermal methods and massage can reduce swelling and increase blood flow to the affected tissues, increasing the amount of oxygen available to damaged hypoxic tissues to promote recovery.^[6]^

Laser therapy can be used in treatment of facial palsy. It is considered a non-invasive and painless therapeutic modality that is appropriate for any type of patient,^[7]^ including those who cannot use corticosteroids, such as diabetic and hypertensive patients. Laser therapy has a favorable prognosis in regeneration of peripheral nerves in both neurosensory and neuromotor deficits, such as trigeminal neuralgia, neuropathy, lower back pain with sciatica, and herpes zoster.^[8]^ Application of laser produces both local and systemic effects that can enhance nerve regeneration.^[9]^ Moreover, laser improves recovery of the injured peripheral nerve and decreases post-traumatic retrograde degeneration of neurons in the corresponding segments of the spinal cord.^[10]^

Research studies have shown that low-level laser therapy (LLLT) increases the functional activity of the injured peripheral nerve, prevents or decreases degeneration in corresponding motor neurons of the spinal cord, and improves axonal growth and myelinization.^[11]^ Recently, high intensity laser therapy (HILT) was introduced to the field of physical therapy and approved by the Food and Drug Administration (FDA) in 2004.^[12]^ The recent development of a high-power pulsed neodymium-doped yttrium aluminum garnet (Nd-YAG) laser (wavelength, 1064 nm), which functions at high peak power, has been shown to induce a non-invasive regenerative therapy capable of reaching and stimulating organs that are difficult to reach with classical lasers, such as large and/or deep areas.^[13]^ Recent studies have described the anti-inflammatory, anti-edemigenic, and antalgic effects of diode laser radiation, justifying its use as therapy for pain and inflammation.

As mentioned above, laser therapy affects tissues differently depending on wavelength, pulse duration, pulse/energy, energy density, and delivery system. Studies of laser dose response suggest that different wavelengths have specific penetration abilities and subsequently different effects on tissue.^[14,15]^ Based on the limited number of studies that have investigated the effect of laser therapy on treatment of facial palsy as well as the existence of new treatment modalities, there is need for further investigation of laser therapy in treatment of acute and chronic facial palsy.

Therefore, the aim of the present study was to investigate and compare the effects of laser therapy in treatment of patients with facial palsy.

## 2 Methods

### 2.1 Study design

We will conduct a systematic review adhering to the reporting guidelines of the preferred reporting items for systematic reviews and meta-analyses (PRISMA) statement.^[16]^

### 2.2 Study registration

This systematic review is registered with PROSPERO (registration number: CRD42020168753; https://www.crd. york.ac.uk/PROSPERO).

### 2.3 Eligibility criteria

#### 2.3.1 Participants

All articles that reported a clinical trial in which human patients with facial palsy were treated with any kind of laser therapy were included. Trials testing other therapies, such as dry needling with or without electrical stimulation or moxibustion (a traditional Chinese method that uses the heat generated by burning herbal preparations containing Artemisia vulgaris, or mugwort) were excluded.

Studies comparing 2 forms of laser therapy and those in which no clinical data were reported were also excluded. No language restrictions were imposed. Dissertations and abstracts were included provided they contained sufficient detail.

#### 2.3.2 Type of interventions

Eligible interventions include any laser-driven therapies specifically designed to manage or treat facial palsy. These treatments could include LLLT, diode laser therapy, or microdose laser therapy of arsenide of Ganglio.

#### 2.3.3 Type of studies

We will include controlled clinical trials published within or after the year 1993 that evaluate the efficacy of laser therapies specifically designed to treat patients with facial palsy. We will exclude historically controlled, quasi-experimental, and single-arm pre-post studies.

#### 2.3.4 Outcome measures

We are primarily interested in outcomes that measure 1 of the following 3 construct domains:

1) Paralysis score
2) Physical facial disability index (PFDI) and social facial disability index (SFDI)
3) Recovery rate of supracordal/infracordal lesions

Other secondary outcomes will be considered depending on review findings; anticipated possibilities include accompanying promotion of mental health.

#### 2.3.5 Language

An English language restriction will be applied.

#### 2.3.6 Publication date and type

Only studies published in peer-reviewed journals will be considered for the systematic review. Studies published between 1993 and 2020 will be considered for review.

### 2.4 Information sources and search strategy

We will design and conduct a search strategy using methods recommended by the Institute of Medicine and will involve several electronic databases: PubMed, Scopus, EBSCO CINAHL, Ovid MEDLINE, Ovid EMBASE, Ovid Cochrane Library, Web of Science, and Ovid PsycINFO. Databases will be searched from January 1, 1993, to the present (maybe June 30, 2020). To maximize the specificity and feasibility of the search, we will focus on Medical Subject Headings (MeSH) such as “laser therapy” or “laser treatment” and “controlled clinical trials,” along with text searches of terms such as ‘facial palsy” and “facial paralysis” (which is a term used by some in referencing a similar construct).

The concept of therapy will be addressed primarily using text words such as “low-level,” “high-level,” and “diode.” The initial electronic search strategy will be supplemented by hand searching the reference lists of eligible studies and by contacting experts in the field to identify any missing, in-progress, or unpublished data. In addition, we will search for reviews on the topic and peruse their reference lists to identify potentially eligible studies that may have been missed through other methods. Finally, clinical trial registries will be searched to identify completed and in-progress studies; if not identified through other methods, the authors will be contacted for details regarding study status. There will be no language restrictions during the initial search (Table 1).

**Table 1:**
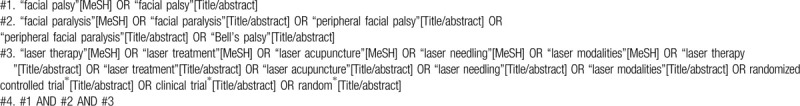
Search strategy for the MEDLINE via PubMed.

### 2.5 Study selection

**Figure 1:**
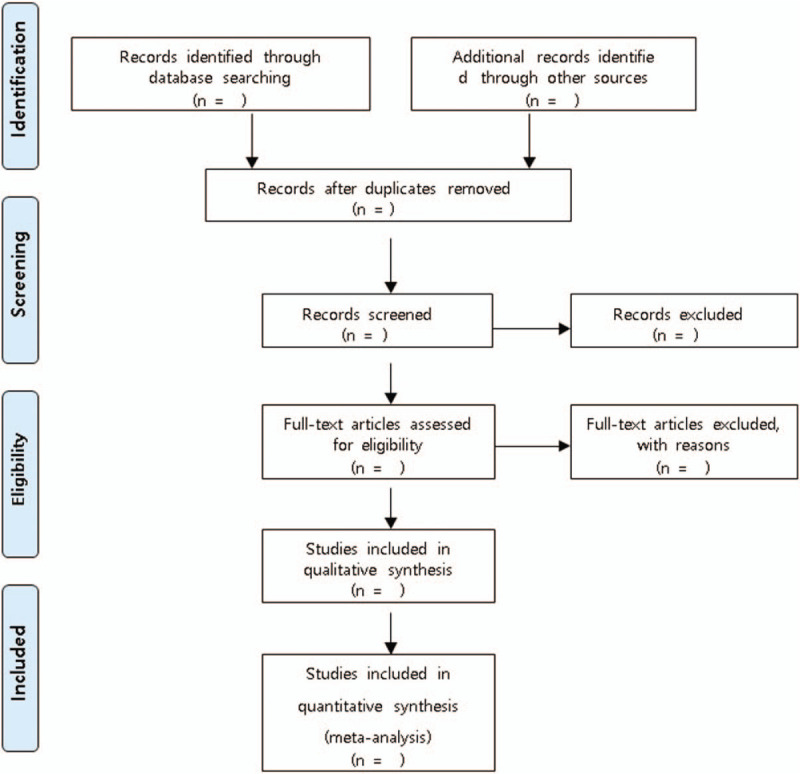
Flow diagram following PRISMA guidelines.

We will upload search results into systematic review software (DistillerSR, Evidence Partners, Ottawa, ON, Canada). In the first round of screening, abstracts and titles will be screened for inclusion. Following abstract screening, eligibility will be assessed through full-text screening. Prior to both abstract and full-text screening, reviewers will undergo training to ensure a basic understanding of the field and purpose of the review. Comprehension of inclusion and exclusion criteria will be assessed through calibration of a small number of studies. Eligibility at both levels (abstract and full-text) will be assessed independently and in duplicate. Any disagreements will be resolved by consensus. If consensus cannot be achieved between the 2 reviewers, a third reviewer will arbitrate (Fig. 1).

### 2.6 Data management

The studies will be managed within the reference management system of EndNoteX9. De-duplication will be facilitated by EndNote also.

### 2.7 Data extraction

Data extractors, working independently, will collect primary data from the included trials using a web-based program (DistillerSR). The extracted data will include patient characteristics, outcomes measured and instruments used, intervention and control characteristics, and factors associated with study quality. Discrepancies in data collection will be adjudicated by consensus.

If data presented in the studies are unclear, missing, or presented in a form that is either un-extractable or difficult to reliably extract, the authors of the study will be contacted for clarification. When data extraction is complete, the authors of the studies will be contacted to ensure accuracy and completeness of data extraction. In addition, the authors of included studies will be asked if they know of any additional studies, either completed or ongoing, that they believe would be eligible for our review.

Author contact will be initiated by email to the corresponding author. If no email address is provided, an internet search will be used to find a current email address; when email address is available, first authors will be carbon copied on all emails to the corresponding author. If emails for the corresponding author are unavailable, corresponding authors will be contacted by telephone.

Authors will be given 1 week to respond to emails, at which time a follow-up email will be sent; if no response is received after an additional 2 weeks, a telephone call will be made to try to contact the author. Attempts to reach authors by telephone will occur for a period of 2 weeks, at which time the author will be classified as uncontactable.

### 2.8 Risk of bias assessment

Primarily, we will use the Cochrane Collaboration's risk of bias tool to evaluate the methodological quality of included studies.^[17]^ If the corresponding study is classified as a non-randomized clinical trial, the methodological quality will be evaluated using RoBINS-I, risk of bias assessment tool used in non-randomized clinical studies.^[18]^

The risk of bias (high/low/unclear) in included studies will be assessed in duplicate by reviewers working independently. Any disagreements will be resolved by consensus; if consensus is unable to be achieved, a third reviewer will arbitrate. Items in the risk of bias assessment will include randomization, quality of randomization (any important imbalances at baseline), allocation concealment, level(s) of blinding/masking, losses to follow-up, intention to treat analysis, handling of missing data, and funding sources.

## 3 Discussion

Facial palsy is a condition that can greatly affect an individual's life by causing pain and cosmetic changes. Treatment with corticosteroid is not necessary in many cases. As a noninvasive external physiotherapy, laser therapy is widely used for treating facial palsy. However, there has been no standardization of frequency or intensity in actual application or establishment of an indication that laser treatment could have a successful therapeutic effect. In recent years, though there have been an increasing number of clinical reports on treatment of facial palsy high-quality trial remain insufficient in number.

This review will begin when necessary trails are meeting. To provide compelling evidence and better guidance in clinic practice, all actions of this review will be performed according to the Cochrane Handbook 5.2.0.

## Author contributions

**Advisory role regarding the interventions as clinical experts:** Byung-Kwan Seo, Yong-Hyeon Baek.

**Analysis and Interpretation of the clinical data:** Bonhyuk Goo, Yeon-Cheol Park.

**Study protocol design:** Sang-Soo Nam, Bonhyuk Goo.

**Writing manuscript:** Jung-Hyun Kim.
